# Exosomal miR‐128‐3p reversed fibrinogen‐mediated inhibition of oligodendrocyte progenitor cell differentiation and remyelination after cerebral ischemia

**DOI:** 10.1111/cns.14113

**Published:** 2023-02-08

**Authors:** Huiqing Hou, Yafei Wang, Lan Yang, Yongjun Wang

**Affiliations:** ^1^ Department of Neurology, Beijing Tiantan Hospital Capital Medical University Beijing China; ^2^ Department of Neurology the Second Hospital of Hebei Medical University Shijiazhuang China; ^3^ China National Clinical Research Center for Neurological Diseases Beijing China; ^4^ Research Unit of Artificial Intelligence in Cerebrovascular Disease, Chinese Academy of Medical Sciences Beijing China; ^5^ Center for Excellence in Brain Science and Intelligence Technology, Chinese Academy of Sciences Beijing China

**Keywords:** exosome, fibrinogen, microRNA, oligodendrocyte progenitor cell, stroke

## Abstract

**Aims:**

To investigate the role of exosomal miR‐128‐3p in promoting fibrinogen‐mediated inhibition of oligodendrocyte progenitor cell (OPC) differentiation and the therapeutic potential of exosomal miR‐128‐3p in cerebral ischemia.

**Methods:**

Mouse models of middle cerebral artery occlusion (MCAO) were established as described previously. MCAO was treated with fibrinogen and exosomes by stereotactically injecting into the left stratum. Mouse cortical OPCs were used for mRNA and miRNA sequencing analysis. Exosomes were isolated from neural stem cells (NSCs) of mice.

**Results:**

Fibrinogen deposition suppressed remyelination after MCAO and inhibited OPC differentiation by activating ACVR1, the bone morphogenetic protein (BMP) signaling type I receptor. In vitro, miR‐sequencing and verification studies revealed that miR‐128‐3p is associated with BMP signaling mediated by ACVR1. Additionally, transfer of NSC‐derived exosomal miR‐128‐3p to OPCs significantly increased myelin basic protein expression and inhibited BMP signaling. Furthermore, NSC‐derived exosomal miR‐128‐3p protected against fibrinogen‐induced demyelination related to BMP signaling, reduced the infarct volume, and improved neurological function after MCAO.

**Conclusions:**

Fibrinogen deposition inhibits remyelination after ischemic damage and NSC‐derived exosomal miR‐128‐3p promotes OPC differentiation into OLs by suppressing BMP signaling, indicating that NSC‐derived exosomal miR‐128‐3p represents a potential therapeutic target for ischemic stroke.

## Graphical abstract

The schematic diagram showed that exosomal miR‐128‐3p reversed fibrinogen‐mediated inhibition of oligodendrocyte progenitor cell differentiation and remyelination after ischemic damage by targeting BMP signaling.

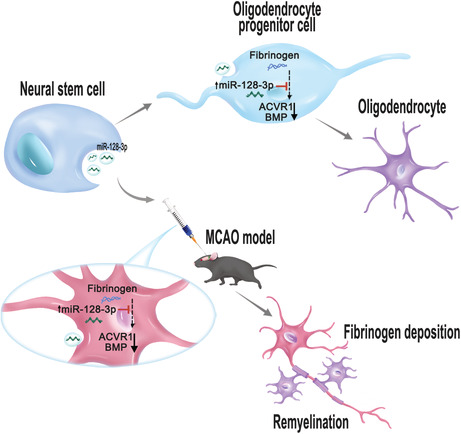

## 1 INTRODUCTION

Fibrinogen is a multifunctional macromolecular protein that could inhibit the repair of brain tissue after vascular damage in addition to promoting coagulation and inflammation.^1^ We recently demonstrated that high fibrinogen levels are associated with poor functional outcome or dependence in ischemic stroke.^2^ Previous studies have also indicated that prevention of fibrinogen deposition is associated with alleviation of infarction and brain edema in stroke models.^3^ However, the underlying molecular mechanism of fibrinogen is unknown. Recent studies have proven that sudden damage to the blood–brain barrier initiates the deposition of fibrinogen in the central nervous system, which can promote astrogenesis and scar formation after cortical injury.^4^, ^5^ Additionally, fibrinogen activates bone morphogenetic protein (BMP) signaling in oligodendrocyte progenitor cell (OPC) and subsequently impedes remyelination.^6^ Importantly, these results reveal that fibrinogen plays a critical role in the inhibition of tissue repair in the central nervous system.^1^

Emerging studies have highlighted that exosomes, as important mediators of intercellular communication, may transfer microRNAs (miRNAs) to target specific cells and modify their functions through cellular pathways.^7^, ^8^ Because the administration of mesenchymal stromal cells (MSC) and that of MSC‐derived exosomes provide equal therapeutic benefits,^9^ exosomes harvested from MSCs have attracted special attention, and their ability to enhance recovery after stroke has been investigated.^10^, ^11^ In addition, exosomes derived from NSCs can also induce brain remodelling^12^ and improve function in a porcine model of ischemic stroke.^13^ Specifically, it has been reported that elevation of miRNA levels in MSC‐derived exosomes promotes axonal growth via regulation of the protein phosphatase and tensin homologue signaling pathway.^14^ Therefore, NSC‐derived exosomes might transfer miRNAs to OPCs, promoting oligodendrocyte (OL) maturation and leading to remyelination after ischemic damage.

Although exosome‐mediated delivery of miRNAs to target cells has been identified as a critical therapeutic strategy for improving neurological functional outcomes in stroke model,^11^, ^15^, ^16^ the mechanism of exosomal miRNA‐mediated OPC differentiation after ischemic stroke remains unknown. In addition, fibrinogen inhibits OPC differentiation and is associated with increased BMP signaling at sites of increased the blood–brain barrier permeability in multiple sclerosis.^6^ Considering the similarity of the blood–brain barrier damage in multiple sclerosis and stroke, miRNA transfection may facilitate OPC differentiation into OLs and accelerate neurorestoration by inhibiting BMP signaling after ischemia.

These findings suggest that fibrinogen may also act as an antagonist after ischemia and that the mechanism of OPC differentiation may be similar in multiple sclerosis and after ischemia. However, the critical role of miRNAs in OPCs and the effect of NSC‐derived exosome transfection after ischemia are largely unknown. In the present study, we assessed whether fibrinogen deposition inhibits remyelination through the BMP signaling after middle cerebral artery occlusion (MCAO). In addition, we performed comprehensive miRNA profiling of OPCs by miRNA‐sequencing analysis and identified that miR‐128‐3p is associated with the impairment of OPC maturation by fibrinogen. Furthermore, we performed in vitro experiments with cultured OPCs to determine the mechanisms involved in the promotion effect of NSC‐derived exosomal miR‐128‐3p transfection on OPC differentiation and evaluate the effect of NSC‐derived exosomal miR‐128‐3p in an MCAO model.

## 2 MATERIALS AND METHODS

### 2.1 Animals

C57BL/6 mice (male, 8–10 weeks) were purchased from Vital River (Beijing, China) and used in this study. The mice were maintained at 22 ± 0.5°C on a 12 h dark/light cycle and provided free access to standard chow and drinking water. Animals were excluded as previously described^17^: the neurological score was no visible neurological deficits or only forelimb flexion, *a* ≤ 75% CBF reduction or ≥60% CBF reperfusion over baseline levels, the histological change was not significant, or more than 20% weight loss. Considering mouse mortality and exclusion, more mice were added to ensure sufficient numbers in the process of experiment.

Mice were randomly divided into six groups: control, MCAO, MCAO + Fibrinogen, MCAO + Ancrod, MCAO + EXO‐miR‐NC, and MCAO + EXO‐miR‐128‐3P. Animals were assigned randomly to each group. All animal studies were approved by the Institutional Animal Care and Use Committee of Beijing Tiantan Hospital and carried out according to the international and national law and policies (ARRIVE guidelines and the Basel Declaration including the 3Rs concept).^18^

### 2.2 Establishment of an MCAO model

A total of 102 mice were subjected to transient focal cerebral ischemia induced by MCAO as previously described.^19^ During surgery, anesthesia was maintained with isoflurane. The left internal carotid was exposed, and a silicone‐coated filament was inserted and advanced through the incised left internal carotid artery until the origin of the middle cerebral artery was blocked. After occlusion for 60 min, the filament was withdrawn to allow reperfusion. Sham‐operated mice underwent the same surgery without MCAO. The mice were sacrificed at 3, 7, and 28 days for further analysis.

Neurological function was evaluated by mNSS assessment, the adhesion removal test, and the rotarod test as described previously.^17^, ^20^ All mice underwent the three neurobehavioral tests at different time points (3, 7, 10, 14, 21, and 28 days) after ischemia and were performed by investigators blinded to the experimental conditions.

### 2.3 Systemic fibrinogen depletion

To investigate fibrinogen deposition and to analyze the effect of fibrinogen on OPC differentiation and remyelination after ischemic damage, fibrinogen was depleted with ancrod.^21^ Ancrod (2 mg, Sigma, California, USA) or control buffer was administered to mice every day via mini‐osmotic pumps (0.5 μL/h) implanted subcutaneously in their backs.^4^

### 2.4 Stereotactic injection and administration pharmacologic inhibitors

To investigate the direct effect of fibrinogen on OPC differentiation and remyelination, mice were anesthetized, and fibrinogen was stereotactically injected into the left stratum 24 h before MCAO as described previously.^22^, ^23^ Fibrinogen (Sigma, California, USA) or albumin (Sigma, California, USA) was dissolved in endotoxin‐free distilled water and diluted to 5 mg/mL with NaCl. Fibrinogen (1 μL of 5 mg/mL) or albumin was slowly injected (0.2 μL/min) with a 10‐μL Hamilton syringe. To analyze the effect of NSC‐derived exosomal miRNAs on MCAO, exosomes (5 μL) were injected into the left stratum in the same way as fibrinogen. To study the effect of BMP signaling inhibition, cultured OPCs were treated with 1 μM DMH1 (Sigma‐Aldrich, MI, USA) before fibrinogen treatment.

### 2.5 Immunofluorescence staining

At different time points after MCAO, mice were deeply anesthetized with pentobarbital sodium and sacrificed by transcardial perfusion with ice‐cold phosphate buffered saline (PBS, pH 7.4) followed by 4% paraformaldehyde in PBS. Coronal sections (25 μm) were taken from the region −1.70 to −2.18 mm relative to Bregma.^24^ After dehydration, the paraffin‐embedded brain sections were washed with PBS and subjected to antigen retrieval with ethylenediaminetetraacetic acid buffer in a microwave. The slices were then permeabilized with 0.3% (v/v) Triton X‐100 in PBS for 20 min, blocked with 5% bovine serum albumin for 20 min, and incubated with primary antibodies against fibrinogen (1:100, Novus, NBP2‐80414), glial fibrillary acidic protein (GFAP; 1:4000, Abcam, Ab4648), ionized calcium binding adapter molecule 1 (Iba1; 1:200, Invitrogen, MA5‐27726), nestin (1:100, Invitrogen, MA1‐110), inhibitor of DNA binding 2 (Id2; 1:1000, Santa Cruz, sc‐398,104), and myelin basic protein (MBP; 1:100, Santa Cruz, sc‐271,524) overnight at 4°C. After being washed in PBS three times, the sections were incubated with FITC‐conjugated goat anti‐rabbit IgG (1:50, Aspen, AS‐1110) and CY3‐conjugated goat anti‐mouse IgG (1:50, Aspen, AS‐1111). The nuclei were counterstained with DAPI (Aspen, AS1075). The regions of interest were visualized with a laser scanning confocal microscope (Olympus IX51). The parameters of the experimental setup for each channel, including the laser power value, HV, gain, and offset were kept constant throughout the imaging process. A colocalization channel was produced for each Z‐stack, and colocalization was quantified in OlyVIA based on overlap. Images of the cortical penumbra L2/3 (−1.70 to −2.18 mm relative to Bregma) of six mice were taken within the ischemic hemispheres at 400× magnification. The mean density was estimated by the Image‐ProPlus 5.1 software.

### 2.6 Measurement of the cerebral infract volume

The brains were removed quickly to assess the infarct volume at 7 and 28 days after MCAO by 2,3,5‐triphenyltetrazolium chloride staining. The brain tissue was cut into eight coronal sections (1 mm thick), which were stained with 2% 2,3,5‐triphenyltetrazolium chloride solution at 37°C for 15 min and fixed with 4% paraformaldehyde. The infarct volume was determined using ImageJ software and calculated with the formula^25^: hemisphere lesion volume (%) = [total infarct volume‐(ipsilateral hemisphere volume – contralateral hemisphere volume)]/contralateral hemisphere volume × 100%.

### 2.7 Enzyme‐linked immunosorbent assay

Brain tissues were cultured with 10 μg/mL MOG35‐55 in RPMI 1640 medium containing 10% fetal bovine serum (FBS) (v/v). The fibrinogen concentration was measured by quantitative enzyme‐linked immunosorbent assay according to the manufacturer's recommendations (ELK Biotechnology, Wuhan, China).

### 2.8 Western blotting

Proteins were extracted from brain tissues and culture OPCs. The protein concentration was measured using a BCA protein assay reagent kit (Aspen, AS1086). As previously described,^17^ the protein lysates were separated by 4–12% Bis‐Tris sodium dodecyl sulfate polyacrylamide gel electrophoresis (SDS‐PAGE; Aspen, AS1012) and transferred to polyvinylidene fluoride (PVDF; Millipore, IPVH00010) membranes. Nonspecific binding sites were blocked by incubation in 5% nonfat dry milk in TBST buffer (20 mmoL/L Tris–HCl, 150 mmoL/L NaCl, and 0.05% Tween 20 (pH 7.5)) for 1 h. The membranes were then incubated at 4°C for 12 h with the following primary antibodies: rabbit anti‐Lef1 (1:1000; Proteintech, 14972‐1‐AP), rabbit anti‐P‐Smad1/5 (1:1000; Cell Signaling Technology, #9516), rabbit anti‐ACVR1 (1:2000; Abcam, ab155981), mouse anti‐MBP (1:1000; Santa Cruz, sc‐271524), and anti‐GAPDH (1:10000; Abcam, ab181602; as a control). After washing in TBST three times, the bound antibodies were detected by incubation with the corresponding secondary antibodies (1:10000; Aspen, AS1107) for 1 h at room temperature. The data were analyzed using an Odyssey Infrared Imaging System (LI‐COR Bioscience, Lincoln, NE, USA), and quantitative analysis was carried out using ImageJ software.

### 2.9 Primary OPC cultures

Mouse cortical OPCs were isolated as previously described with slight modifications.^26^ Briefly, the brains of C57BL/6 mouse pups (within 24 h of birth) were quickly harvested on ice. After removal of the olfactory bulb, basal nucleus, hippocampus, meninges, and blood vessels, the cerebral cortices were dissected and cut into approximately 1 mm^3^ pieces in D‐Hank's solution and then transferred to culture medium (DMEM/F12 containing 25 μg/mL insulin, 100 μg/mL Apo‐transferrin, 20 nM progesterone, 60 mM putrescine, 30 nM sodium selenite, 20 ng/mL bFGF, and 20 ng/mL EGF). The cells were gently isolated by mechanical pipetting until there were no clumps or only small clumps in the cell suspension. After centrifugation (300 g, 5 min), the supernatant was discarded and resuspended in fresh culture medium. After being passed through a 200‐mesh cell sieve, the cells were cultured in a humidified incubator at 37°C in 5% CO_2_ until they reached confluence (7–10 days). To remove microglia, glia‐containing flasks were shaken at a speed of 180 r/min for 1 h. The OPCs were then shaken at a speed of 200 r/min overnight. The OPCs were cultured in basic medium for 3 to 5 days. Half of the medium was exchanged every 2 days. After 2 weeks, the cell balls were collected by centrifugation (300 g, 5 min) and digested with 0.25% trypsin at 37°C for approximately 5 min, and then the digestion was terminated with OPC culture medium (DMEM/F12 containing 4 mM l‐glutamine, 1 mM sodium pyruvate, 0.1% bovine serum albumin, 50 μg/mL Apo‐transferrin, 5 μg/mL insulin, 30 nM sodium selenite, and 10 nM D‐biotin, 10 nM hydrocortisone, 10 ng/mL PDGF‐AA, and 10 ng/mL bFGF). To simulate the ischemia, OPCs were incubated under oxygen–glucose deprivation (OGD) conditions as described previously.^27^ To examine the role of fibrinogen in OPC maturation, primary OPCs were treated with fibrinogen at a concentration of 2.5 mg/mL or vehicle. Double immunostaining of the mature OL marker MBP was performed after treatment.

### 2.10 Isolation and enrichment of mouse NSCs

NSCs were isolated from the cortical tissues of fetal mice as described previously with slight modifications.^28^ Briefly, cortical tissues were removed from embryonic day (E) 13.5 C57BL/6 mice and quickly harvested on ice. After digestion with 0.25% trypsin‐ethylenediaminetetraacetic acid and mechanical isolation, the tissue pellet was filtered at room temperature. The cells were cultured in medium (DMEM/F12 containing 25 μg/mL insulin, 100 μg/mL Apo‐transferrin, 20 nM progesterone, 60 mM putrescine, 30 nM sodium selenite, 20 ng/mL bFGF, and 20 ng/mL EGF). Primary neurospheres were collected, centrifuged, and replated. After three rounds of neurosphere formation, enriched NSCs were harvested.

### 2.11 RNA expression

Total RNA was extracted from brain tissues or OPCs using TRIzol reagent (Invitrogen, California, USA) following previously described methods.^29^ The integrity of the RNA was confirmed by 1.5% agarose gel electrophoresis. Finally, an EnTurbo™ SYBR Green PCR SuperMix (ELK Biotechnology, Wuhan, China) was used to quantify qualified RNA concentrations through Qubit 3.0.

Total RNA (2 μg) was used for stranded RNA sequencing library preparation using the KC™ Stranded mRNA Library Prep Kit for Illumina® (Catalogue No. DR08402, Wuhan Seqhealth Co., Ltd. China) according to the manufacturer's protocol. PCR products corresponding to 200–500 bps were enriched, quantified, and finally sequenced on a HiSeq X‐10 sequencer (Illumina).

### 2.12 RNA‐Seq data analysis

Raw RNA data were mapped to the *Mus musculus* reference genome (GRCm39; ftp://ftp.ncbi.nih.gov/genomes/M‐musculus/) using STAR software (version 2.5.3a) with default parameters. Reads mapped to the exon regions of each gene were counted by featureCounts (Subread‐1.5.1; Bioconductor), and then RPKMs were calculated. After all relevant correction and normalization steps were performed, statistical tests were applied to identify differentially expressed RNAs. rMATS (version 3.2.5) was used to detect alternative splicing events with a cutoff of 0.05 and absolute value of Δψ of 0.05.

### 2.13 miRNA expression

Total RNA was extracted from OPCs using TRIzol reagent (ELK Biotechnology, EP013) as described above. Then, the total RNA was used as input for miRNA library preparation by using the KC‐Digital™ small RNA Library Prep Kit for Illumina® (Catalogue No. DR08602, Wuhan Seqhealth Co., Ltd. China) following the manufacturer's protocol. This kit is used to eliminate duplication bias in the PCR and sequencing steps. After the eluted cDNA library was separated, ~160 bp bands were isolated, purified, and quantified by Thermo Fisher Qubit 3.0 and finally sequenced on a HiSeq X‐10 sequencer (Illumina) with a PE150 model.

### 2.14 miRNA‐Seq data analysis

Raw sequencing data were first subjected to quality assessment and then filtered using the fastx toolkit (version: 0.0.13.2) to remove low‐quality sequences, and adapter sequences were trimmed by cutadapt (version: 1.15). Clean reads were further processed by in‐house scripts to eliminate duplicate bias introduced during library preparation and sequencing. The deduplicated sequences from each sample were mapped to the reference genome (GRCm39; ftp://ftp.ncbi.nih.gov/genomes/M‐musculus/) using the Bowtie (version: 1.1.2). The mirdep2 package (version: 2.0.0.8) was used to map the reads to the known major miRNAs in the miRBase database and predict new miRNAs. Differences in miRNA expression between groups were determined using the edgeR package (version: 3.12.1). A cut‐off of the *p* < 0.05 and |Log_2_Fold‐change| > 1 was used to identify miRNA with significantly differences in expression. A miR‐mRNA coexpression network was constructed by WGCNA (version: 1.61), with a correlation threshold >0.8 between miRNAs and mRNAs.

### 2.15 Quantitative real‐time PCR (qRT‐PCR) analysis

Brain tissues and cultured cells were prepared. Briefly, total RNA was digested with DNase 1. The RNA was reverse transcribed into cDNA using the M‐MLV Reverse Transcription Synthesis Kit (ELK Biotechnology, Wuhan, China), and the relative expression levels of miRNA were determined using the EntiLink™ 1st Strand cDNA Synthesis Kit (ELK Biotechnology, Wuhan, China) following the manufacturer's instructions. Relevant primers used to measure gene expression on the StepOne™ real‐time PCR system (Life Technology, CA, USA) or QuantStudio 6 Flex PCR system (Life Technology, CA, USA) are listed in Table S1. Initial incubation was performed at 95°C for 10 min, followed by 40 cycles of 95°C for 15 s and 60°C for 60 s. The data were evaluated by Sequence Detection Systems software. The data were analyzed using the 2^−ΔΔCT^ method.

### 2.16 Synthesis of miR‐128‐3P mimics and cell transfection

miR‐128‐3P mimic (5’‐TCACAGTGAACCGGTCTCTTT‐3′) and miR‐NC as a negative control were synthesized by RiboBio (Guangzhou, China). NSCs/OPCs were transferred to a 24‐well culture plate containing an appropriate amount of complete medium to achieve a cell density 30%–50%. Next, NSCs/OPCs were cultured in serum‐free Opti‐MEM (Gibco, China) containing 10% FBS (Gibco, 10,099,141) and 1% penicillin/streptomycin (Gibco, 15,140,122). Then, NSCs/OPCs were transfected with 50 nmol miR‐128‐3P mimic, or negative control using Lipofectamine 2000 (Invitrogen) according to the manufacturer's instructions. After transfection, NSCs/OPCs were cultured in vitro at 37°C for 24 h, and the transfected NSCs/OPCs were treated with fibrinogen.

### 2.17 Isolation of exosomes

Exosomes were isolated from the culture medium of NSCs as previously described.^30^ Briefly, NSCs were plated in poly‐l‐ornithine‐coated (100 μg/mL, Sigma, P4957) dishes and cultured in NSC medium (DMEM/F12, HyClone, SH30023) for 48 h. The culture supernatant was collected, Total Exosome Isolation reagent (Invitrogen, 4,478,359) was added, and the sample was filtered through a 0.22‐μm strainer and centrifuged in a subultraspeed low‐temperature centrifuge at 100,000 g for 2 h. After that, the supernatant was discarded, and the pellets were resuspended in 1× PBS and preserved at −80°C for later use.

### 2.18 Nanosight tracking analysis

The size and concentration of exosomes were evaluated at VivaCellBiosceinces by PMX 110 ZetaView nanosight tracking analysis system (Particle Metrix, Meerbusch, Germany) and ZetaView 8.04.02 SP2 software. The polystyrene particles were used to standardize the ZetaView system.

### 2.19 Labeling of exosomes with PKH26

Cells were stained with phalloidin‐FITC (green, Yeasen, 40735ES75), and exosomes were labeled with PKH26 (red, MKBio, MX4021). A fluorescence microscope (Olympus IX51) was used to obtain the images of the PKH26‐labeled exosomes.

### 2.20 Transmission electron microscopy

Negative staining of exosome suspensions followed by transmission electron microscopy was used to determine the vesicle shape and size distribution. Aliquots of exosome suspensions were dispensed on sheets of Parafilm in a humidified petridish, and the vesicles were deposited on a carbon‐coated grid (300 mesh) for 3 min. Subsequently, the grid was negatively stained with 20 drops of filtered 1% uranyl acetate for 3 min, and the excess staining solution was blotted off. The droplets of exosomes were removed with filter paper and air‐dried at RT. The exosomes were visualized with a transmission electron microscope (JEM‐1230, JEOL).

In a subset of mice, the structure of the myelin sheath was observed at the ultrastructural level by transmission electron microscopy. Mice were deeply anesthetized and perfused with 4% paraformaldehyde and 2.5% glutaraldehyde in PBS. The harvested brain samples were placed in cold fixative solution with consisting of 2.5% glutaraldehyde in PBS (0.1 M, pH = 7.4) at 4°C for 24 h. The brains were cut into small pieces along the midline and oriented to obtain cross‐sections of the axons. After washing with PBS, the samples were postfixed in 1% osmium tetroxide solution for 1 h. The samples were then washed again using PBS. The tissues were dehydrated in a series of ascending ethanol solutions and permeated with propylene oxide. Then, the samples were embedded with pure Epon resin and incubated at 60°C for 72 h. Ultrathin serial sections of the center of the embedded blocks containing the axons were prepared using an ultramicrotome (thickness = 60 nm). The slices were then placed on a copper grid and stained with 2% uranyl acetate and lead citrate. All sections were examined under a JEM‐1230 transmission electron microscope at 80 kV.

### 2.21 Statistical analysis

The data are presented as the means ± SEM. GraphPad Prism software was used for statistical analysis. Each experiment was conducted three times independently. Differences in continuous variables with normal distributions between two experimental groups were examined by unpaired Student's *t*‐test. Statistical significance among multiple groups was tested by one‐way ANOVA with Student Newman Keuls or Dunn's *T*‐tests. Continuous variables with non‐normal distributions were analyzed by the Mann–Whitney *U* rank sum test. In all analyses, *p* < 0.05 was considered statistically significant.

## 3 RESULTS

### 3.1 Fibrinogen deposition in the ischemic boundary zone after ischemia

**FIGURE 1:**
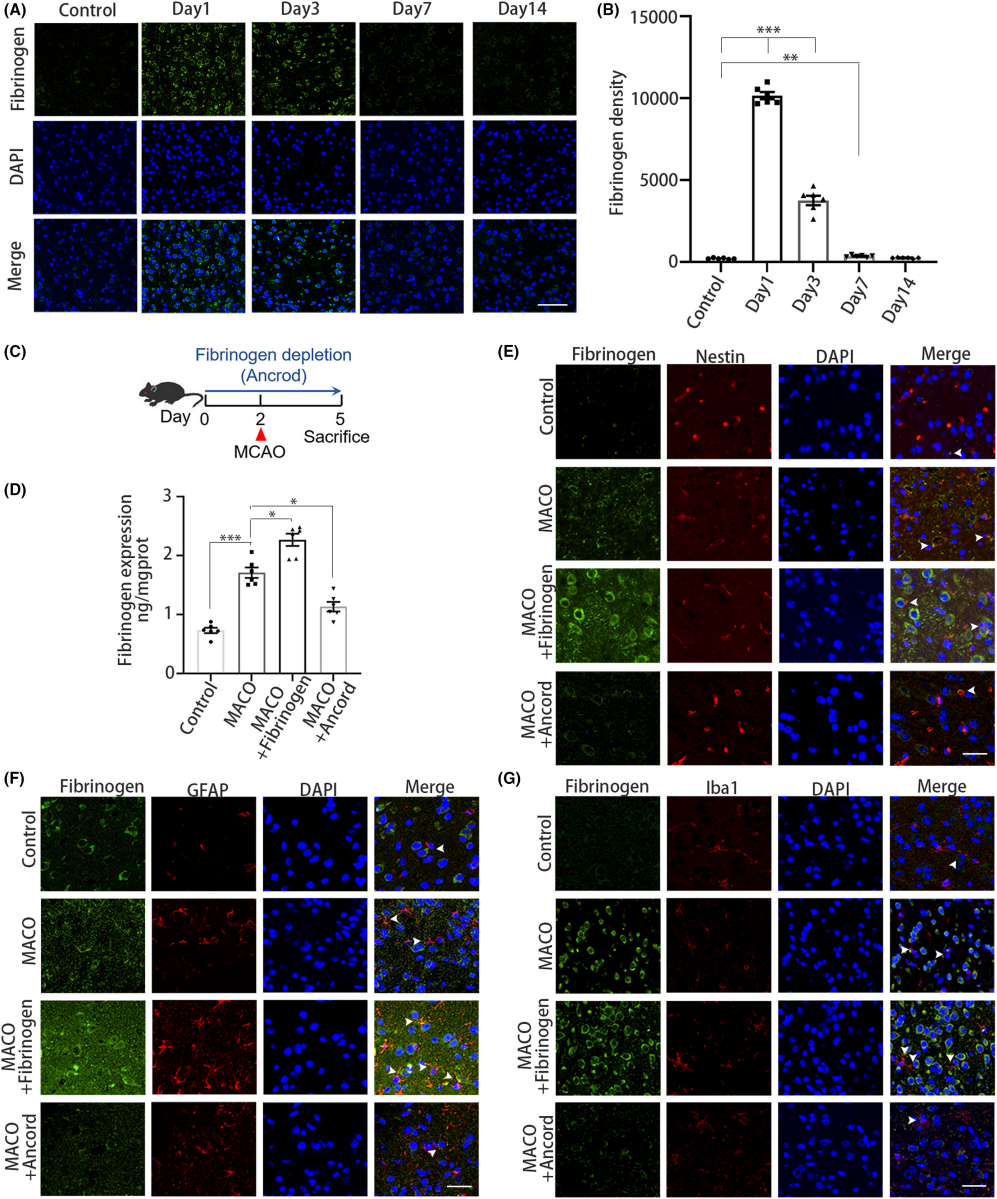
Fibrinogen deposition after ischemia. (A) Fibrinogen immunostaining (green) in the peri‐infarct areas at 1, 3, 7, and 14 days after MCAO and uninjured mice. Scale bar, 50 μm. (B) Quantification of fibrinogen immunoreactivity in the peri‐infarct areas per area. (C) Scheme illustrating MCAO on ancrod‐administered mice. (D) Enzyme‐linked immunosorbent assay quantification of fibrinogen. (E) Nestin (red) and fibrinogen (green) immunostainings in the peri‐infarct areas 3 days after MCAO. White arrowheads show the colocalization of nestin and fibrinogen. Scale bar, 25 μm. (F) GFAP (red) and fibrinogen (green) immunostainings in the peri‐infarct areas 3 days after MCAO. White arrowheads show the colocalization of GFAP and fibrinogen. Scale bar, 25 μm. (G) Iba1 (red) and fibrinogen (green) immunostainings in the peri‐infarct areas 3 days after MCAO. White arrowheads show the colocalization of Iba1 and fibrinogen. Scale bar, 25 μm. *N* = 6 mice. Data are presented as mean ± SEM, one‐way ANOVA, *p < 0.05, **p < 0.01, ***p < 0.001.

Since fibrinogen has been observed in the lesion core and subventricular zone after ischemia,^4^ we investigated fibrinogen deposition around the peri‐infarct areas. The data demonstrated that fibrinogen was deposited 1, 3, 7, and 14 days after MCAO (Figure 1A). The results revealed that fibrinogen levels were significantly increased 1, 3, and 7 days after ischemia (Figure 1B).

To further quantify fibrinogen expression after stroke onset, we examined fibrinogen levels at 3 days post‐ischemia. Stereotactic injection of fibrinogen resulted in a significant increase in fibrinogen levels, while fibrinogen levels were robustly reduced in the group in which fibrinogen was depleted with the pharmacologic reagent ancrod (Figure 1C, D). Fibrinogen has been shown to be a marker of the blood–brain barrier permeability,^31^ as it may regulate cell activity in the context of ischemia. To examine whether fibrinogen deposition occurs specifically after ischemia, we investigated whether fibrinogen surrounded Nestin^+^ neurons, GFAP^+^ astrocytes, and Iba1^+^ microglia after MCAO (Figure 1E–G). Stereotactic injection of fibrinogen led to increased fibrinogen deposition surrounding neurons, astrocytes, and microglia. Systemic fibrinogen depletion with ancrod led to a robust reduction in fibrinogen deposition. Overall, these results show that ischemic damage results in fibrinogen deposition into the boundary zone, potentially implying that fibrinogen overexpression might alter the brain microenvironment.

### 3.2 Fibrinogen inhibits remyelination and disrupts OPC differentiation by activating ACVR1

**FIGURE 2:**
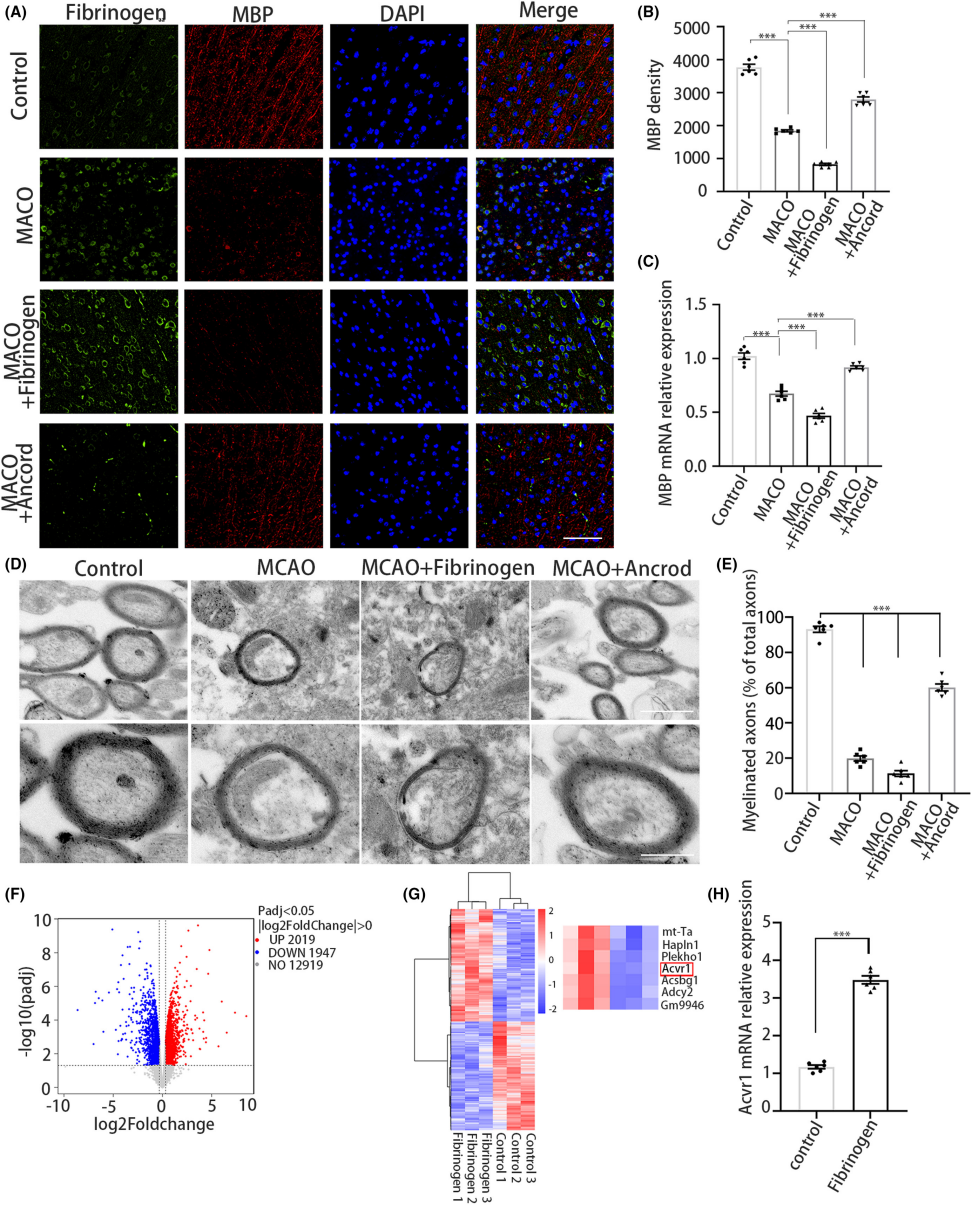
Fibrinogen inhibits remyelination after MCAO and disrupts OPC differentiation by activating ACVR1. (A) MBP (red) and fibrinogen (green) immunostaining in the peri‐infarct areas 7 days after MCAO. Scale bar, 50 μm. (B) Quantification of MBP immunoreactivity in the peri‐infarct areas per area. *N* = 6 mice. (C) MBP mRNA expression. *N* = 6 mice. (D) Transmission electron microscope analysis was performed on the peri‐infarct areas. Scale bar, 1 μm (top), 0.5 μm (bottom). (E) Quantification of the myelinated axons (% of total axons). *N* = 6 mice. (F) Volcano plot of differentially expressed genes. Red points represent upregulated genes, blue points represent downregulated genes, and gray points represent the unchanged genes. (G) Heatmap of differentially expressed genes in untreated OPCs and fibrinogen‐treated OPCs. *N* = 3 per group. (H) The verification of ACVR1 mRNA expression. *N* = 6 per group. Data are presented as mean ± SEM, one‐way ANOVA, or unpaired Student's *t*‐test, ***p < 0.001.

As fibrinogen has been shown to be necessary for OPC differentiation and myelination in demyelinating diseases,^6^, ^32^ but not after ischemic damage, we investigated fibrinogen deposition related to MBP^+^ OL distribution after ischemia. Fibrinogen and MBP double‐staining revealed that fibrinogen depletion with the pharmacologic reagent ancrod increased MBP expression after MCAO (Figure 2A–C). These results imply that fibrinogen deposition led to failure of OPCs to differentiate into OLs after MCAO. In addition, we analyzed the myelin structure in the peri‐infarct areas. Figure 2D, E shows that fibrinogen deposition impeded myelin repair at 14 days, revealing that fibrinogen overexpression resulted in the suppression of remyelination after MCAO. To evaluate the damaging impact on myelinated axons, we calculated the ratio of axon diameter to myelinated fiber diameter (*g*‐ratio). The g‐ratio was lower in the fibrinogen‐treated MCAO than that in ancrod‐treated MCAO (Figure S1). These results might indicate that fibrinogen facilitated the damaging impact on myelin and axon.

Whole‐genome microarray analysis of OPCs treated with fibrinogen was performed in the present study to identify the mechanism by which fibrinogen affects OPC differentiation. There were 2019 upregulated genes and 1947 downregulated genes (Figure 2F). Gene ontology enrichment analysis of the differentially expressed genes in untreated OPCs and fibrinogen‐treated OPCs was performed. According to the heatmap, ACVR1 expression was upregulated in fibrinogen‐treated OPCs (Figure 2G). To verify the differential expression of ACVR1 revealed by RNA‐sequencing, we assessed ACVR1 mRNA expression. The results showed that relative ACVR1 mRNA expression was significantly increased in OPCs after treatment with fibrinogen (Figure 2H). Taken together, these data indicate that fibrinogen disrupts OPC differentiation by activating ACVR1.

### 3.3 miR‐128‐3p mediates OPC differentiation by targeting fibrinogen‐BMP signaling

**FIGURE 3:**
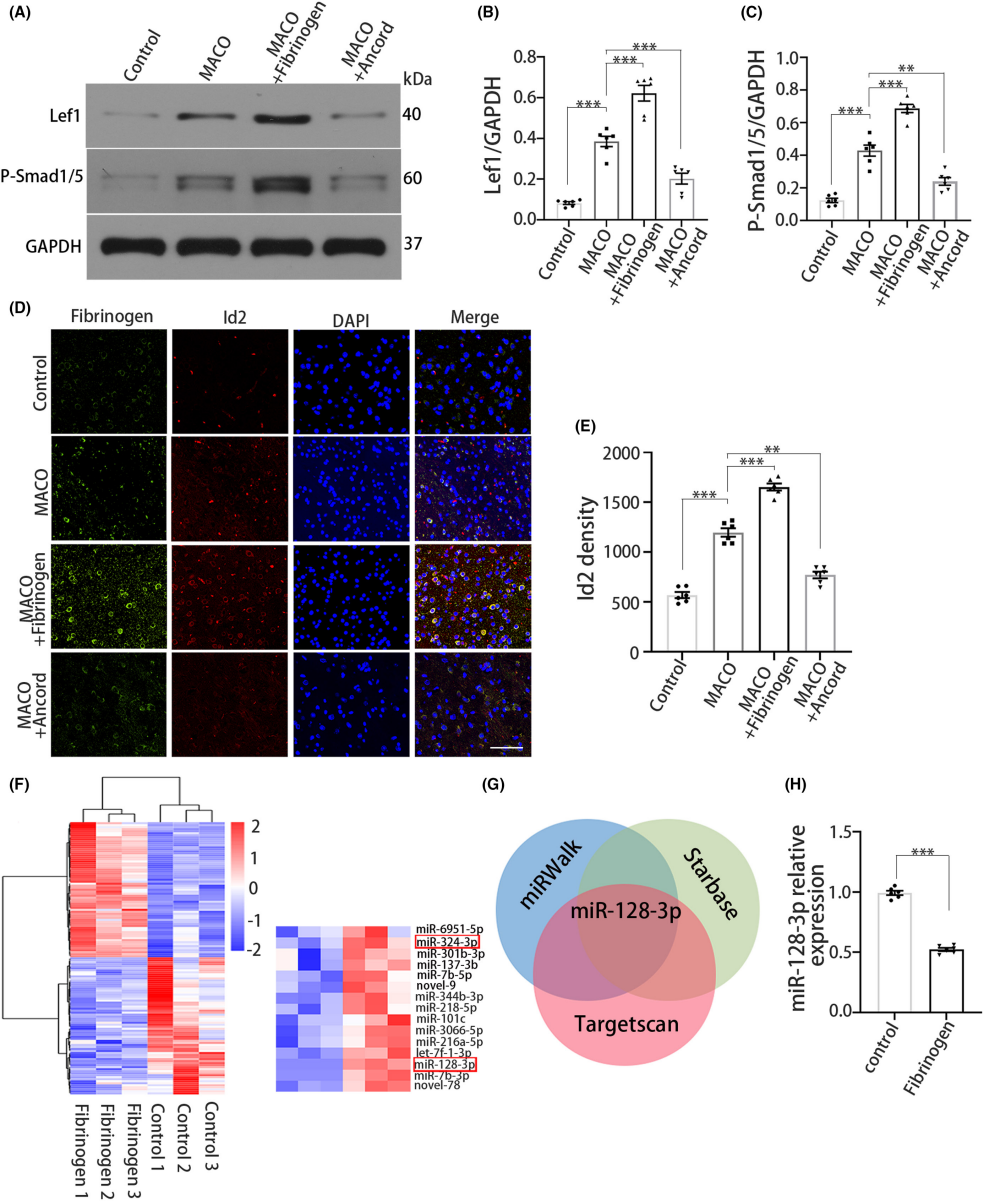
miR‐128‐3p mediates OPC differentiation by targeting fibrinogen‐BMP signaling. (A) Immunoblot analysis for Lef1 and P‐Smad1/5 after MCAO treated with fibrinogen or ancrod. (B and C) Quantification of Lef1 and P‐Smad1/5 expression. *N* = 6 mice. (D) Id2 (red) and fibrinogen (green) immunostaining. Scale bar, 50 μm. (E) Quantification of Id2 immunoreactivity. *N* = 6 mice. (F) Comparison of miRNAs expression in fibrinogen‐treated OPCs and untreated OPCs by miRNAs sequencing. *N* = 3 per group. (G) Venn diagram displaying miR‐128‐3p computationally predicted targets by three different prediction algorithms: targetScan, starbase, and miRWalk. (H) The verification of miR‐128‐3p relative expression by qRT‐PCR analysis. *N* = 6 per group. Data are presented as mean ± SEM, one‐way ANOVA or unpaired Student's *t*‐test, **p < 0.01, ***p < 0.001.

To identify the molecular mechanisms underlying the effect of fibrinogen on OPC differentiation, we measured Lef1 expression, which is regulated by ACVR1 and associated with OPC maturation.^6^, ^33^ Since previous studies have reported that fibrinogen treatment increases Lef1 expression in OPCs,^6^ we used an in vitro model of ischemia induced by OGD. Fibrinogen treatment increased Lef1 expression in OGD OPCs. In contrast, an inhibitor of the BMP type I receptor ACVR1, DMH1, blocked the fibrinogen‐induced change in Lef1 and Id gene expression in OPCs after OGD (Figure S2A‐E). In vivo, stereotactic injection of fibrinogen increased the protein expression of Lef1 and induced Smad1/5 phosphorylation (P‐Smad1/5) (both of these proteins are transcriptional mediators of BMP signaling). In addition, fibrinogen depletion with ancrod reduced the expression of Lef1 and P‐Smad1/5 (Figure 3A–C). Using immunofluorescence staining, we confirmed that fibrinogen injection resulted in increased expression of the downstream transcriptional mediator Id2 and that fibrinogen depletion resulted in a reduction in Id2 expression (Figure 3D, E). These results indicate that fibrinogen activates BMP signaling in OGD‐treated OPCs and in MCAO.

Given that ACVR1 is a key marker of stem cells and that its expression is regulated by miRNAs,^34^ we explored whether fibrinogen modulates the differential expression of miRNAs during OPC differentiation. The miRNA targets of ACVR1 were screened, and miRNA‐128‐3p, miRNA‐342‐3p, and miR‐344g‐5p were predicted as targets using miRanda and RNAhybrid. We compared the miRNA expression profile of cultured OPCs treated with fibrinogen with that of untreated OPCs by microarray analysis. The results revealed that the expression of 66 miRNAs was upregulated and that the expression of 70 miRNAs was downregulated in fibrinogen‐treated OPCs. Among these miRNAs, the expression of miRNA‐128‐3p and miRNA‐342‐3p was significantly downregulated in fibrinogen‐treated OPCs (Figure 3F). Then, using TargetScan, tarBase, and miRWalk, miRNA‐128‐3p was identified as the most likely target of ACVR1 (Figure 3G). qRT‐PCR analysis confirmed that miRNA‐128‐3p expression was downregulated in fibrinogen‐treated OPCs (Figure 3H), implying that miRNA‐128‐3p acts as a direct mediator of OPC differentiation by targeting fibrinogen‐BMP signaling.

### 3.4 Transfection of NSC‐derived exosomes with miR‐128‐3p

**FIGURE 4:**
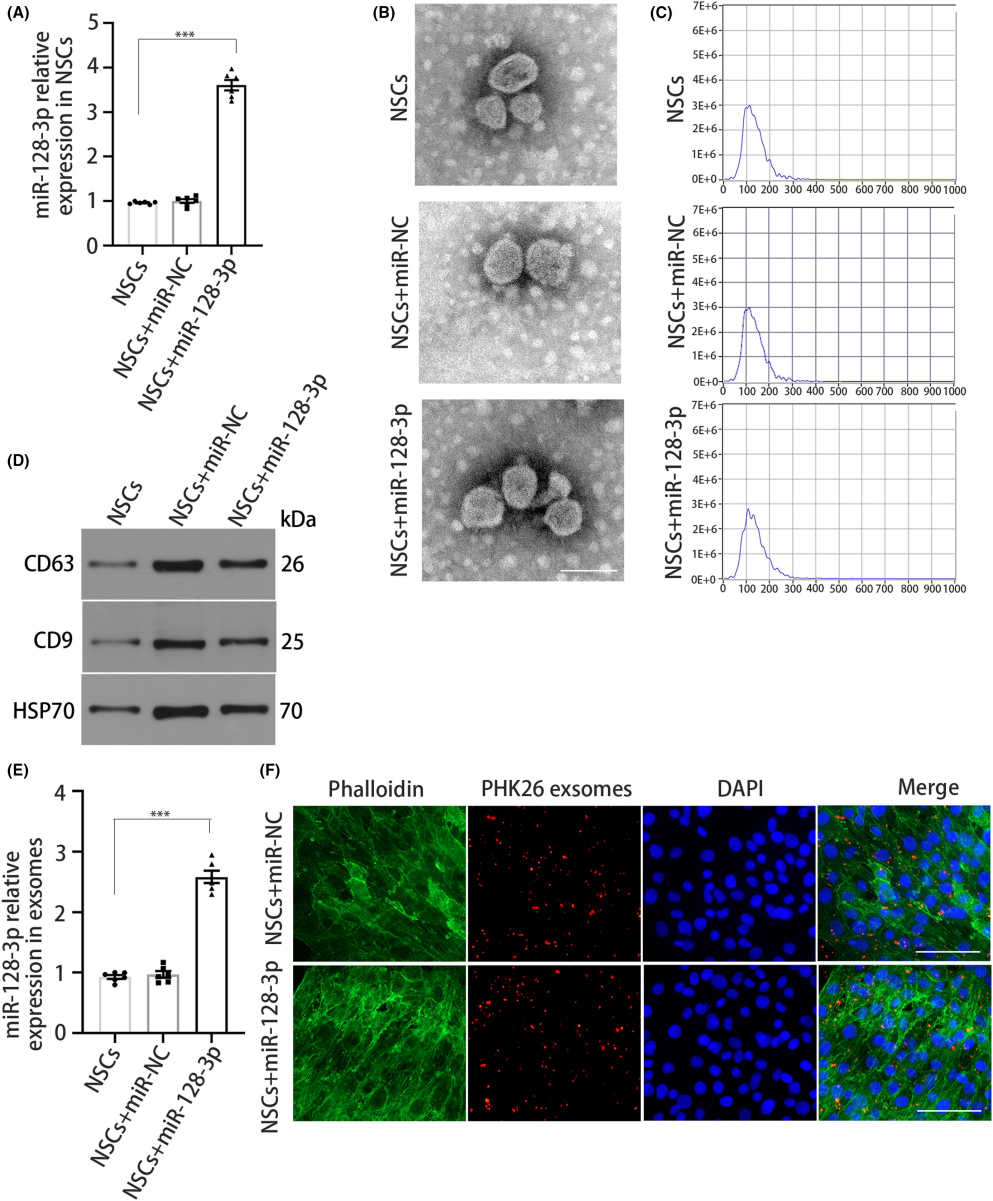
NSC‐derived exosomes infect with miR‐128‐3p. (A) miR‐128‐3p expression in NSCs. (B) Transmission electron microscope analysis for NSCs secreted exosomes. Scale bar, 100 nm. (C) Nanosight tracking analysis for exosome size distribution and number. (D) Immunoblot for levels of exosomal markers, HSP70, CD9, and CD63. (E) miR‐128‐3p expression in exosomes. (F) Confocal microscopy for internalization of PKH26‐labeled exosomes (red). Fluorescein phalloidin‐FITC (green) was used to label F‐Actin, while DAPI (blue) was used to label nuclei. Scale bar, 50 μm. *N* = 6 per group. Data are presented as mean ± SEM, unpaired Student's *t*‐test, ***p < 0.001.

To determine the biological function of miR‐128‐3p in NSCs, we transfected NSCs with miR‐128‐3p mimics or miR‐NC. qRT‐PCR analysis confirmed that miR‐128‐3p was expressed in NSCs (Figure 4A). Next, exosomes from NSCs were isolated by ultracentrifugation. Transmission electron microscopy and nanosight tracking analysis were used to determine the size and shape of the exosomes (Figure 4B, C). The expression of the typical exosomal markers HSP70, CD9, and CD63 was assessed (Figure 4D). In addition, we confirmed that relative miR‐128‐3p expression in exosomes harvested from miR‐128‐3p‐transfected NSCs was significantly higher than that in exosomes harvested from miR‐NC‐transfected NSCs (Figure 4E).

To verify whether exosomes can transfer miR‐128‐3p to OPCs, we performed coculture experiments to observe the internalization of exosomes by OPCs. PHK26‐labeled exosomes derived from NSCs enriched with miR‐128‐3p were incubated with fibrinogen‐treated OPCs. Then, the uptake of exosomes was investigated after 24 h of cocultivation. Many exosomes were invested by recipient cells (Figure 4F), suggesting that metastatic NSC‐derived exosomes can be taken up by recipient OPCs.

### 3.5 NSC‐derived exosomal miR‐128‐3p promotes OPC differentiation

**FIGURE 5:**
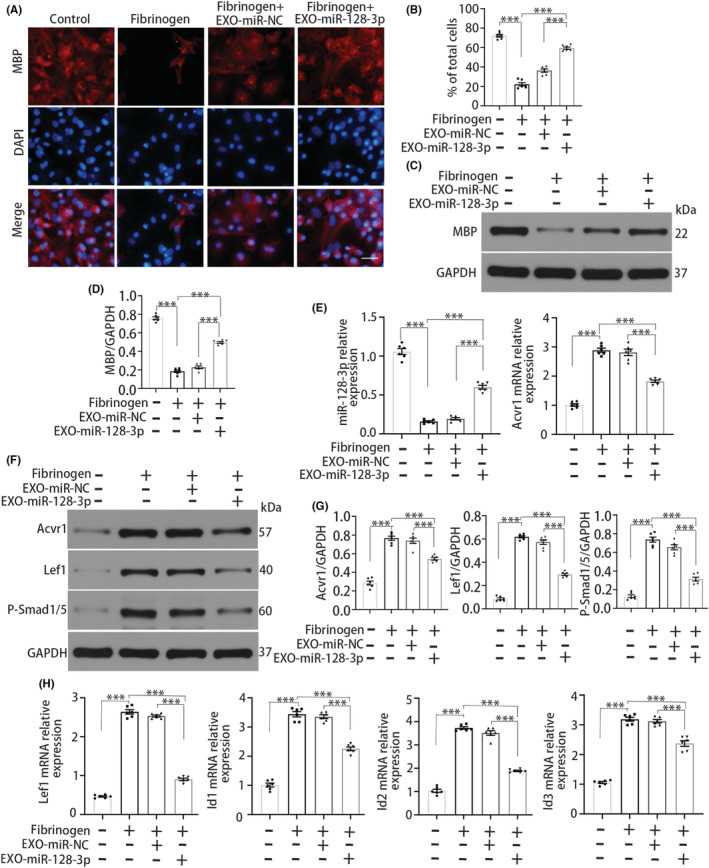
NSC‐derived exosomal miR‐128‐3p promotes OPC differentiation. (A) MBP (red) immunostaining in OPCs. Scale bar, 20 μm. (B) MBP^+^ cells of total cells. (C) Immunoblot analysis for MBP in OPCs. (D) Quantification of MBP. (E) qRT‐PCR analysis for miR‐128‐3p and ACVR1 expression in OPCs. (F) Immunoblot analysis for ACVR1, P‐Smad1/5, and Lef1 in OPCs. (G) Quantification of ACVR1, P‐Smad1/5, and Lef1 expression. (H) Lef1, Id1, Id2, and Id3 mRNA expression in OPCs. *N* = 6 per group. Data are presented as mean ± SEM, one‐way ANOVA, ***p < 0.001.

To investigate the role of NSC‐derived exosomal miR‐128‐3p in the differentiation of fibrinogen‐treated OPCs, OPCs were cocultured with NSC‐derived exosomal miR‐128‐3p. Fibrinogen treatment decreased MBP expression in OPCs, while NSC‐derived exosomal miR‐128‐3p treatment increased MBP expression in fibrinogen‐treated OPCs (Figure 5A–D). The results suggest that NSC‐derived exosomal miR‐128‐3p promotes OPC differentiation into MBP^+^ OLs, which is inhibited by fibrinogen. Next, miR‐128‐3p expression was determined, and it was found that fibrinogen treatment downregulated miR‐128‐3p expression (Figure 5E). Furthermore, NSC‐derived exosomal miR‐128‐3p treatment reversed the change in ACVR1 expression in fibrinogen‐treated OPCs, indicating that exosomal miR‐128‐3p directly antagonizes the effect of fibrinogen by inhibiting ACVR1 (Figure 5E–G).

To further determine whether miR‐128‐3p promotes the differentiation of fibrinogen‐treated OPCs by mediating ACVR1, we examined Lef1 expression. Fibrinogen treatment increased Lef1 expression in OPCs, while Lef1 expression was decreased in fibrinogen‐treated OPCs treated with NSC‐derived exosomal miR‐128‐3p (Figure 5F–H). We next sought to identify the transcriptional mediators of BMP signaling, which is involved in the fibrinogen‐mediated inhibition of OPC differentiation into MBP^+^ OLs.^6^ Fibrinogen treatment upregulated the expression of P‐Smad1/5, Id1, Id2, and Id3 (Figure 5E–G). In contrast, exosomal miR‐128‐3p treatment significantly reduced the expression of these transcriptional mediators, suggesting that NSC‐derived exosomal miR‐128‐3p antagonizes the effect of fibrinogen by blocking BMP signaling and promotes OPC differentiation.

To determine whether NSC‐derived exosomal miR‐128‐3p is necessary for the modulation of ACVR1 activation and OPC differentiation in an in vitro model of ischemia, we treated OPCs with OGD. Treatment of OGD OPCs with NSC‐derived exosomal miR‐128‐3p promoted Lef1 and P‐Smad1/5 expression in fibrinogen‐induced OPCs (Figure S3A, B) and facilitated OPC differentiation into MBP^+^ OLs (Figure S2C, D), suggesting that exosomal miR‐128‐3p promoted OGD OPC differentiation by suppressing fibrinogen‐induced BMP receptor activation.

### 3.6 NSC‐derived exosomal miR‐128‐3p targets BMP signaling

**FIGURE 6:**
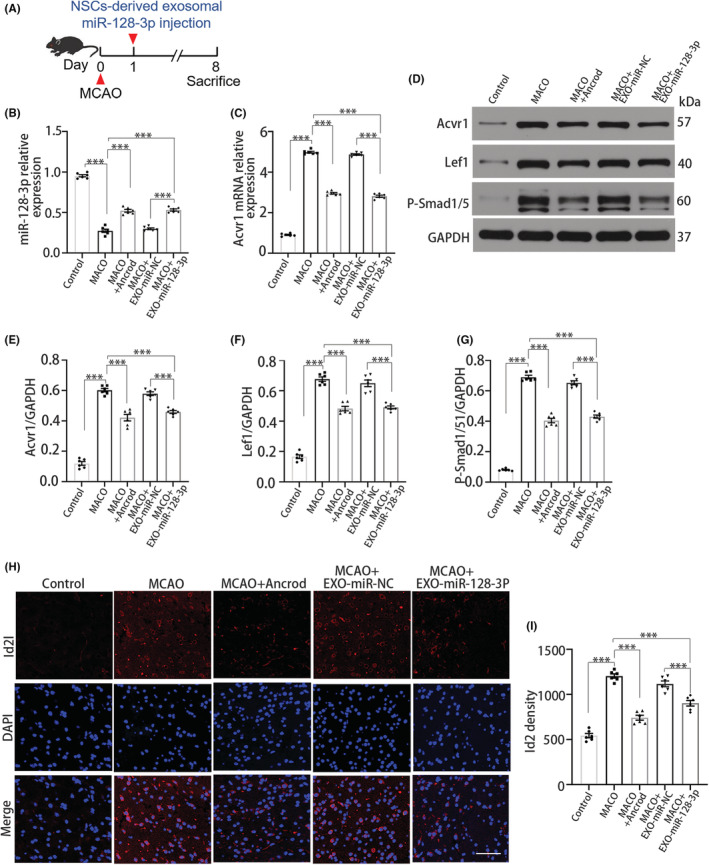
NSC‐derived exosomal miR‐128‐3p targets BMP signaling in MCAO. (A) Scheme illustrating MCAO on NSC‐derived exosomal miR‐128‐3p‐administered mice. (B) qRT‐PCR analysis for miR‐128‐3p expression. (C) qRT‐PCR analysis for ACVR1 expression. (D) Immunoblot analysis for ACVR1, Lef1, and P‐Smad1/5. (E–G) Quantification of ACVR1, Lef1, and P‐Smad1/5 expression. (H) Id2 (red) immunostaining. Scale bar, 50 μm. (I) Quantification of Id2 immunoreactivity. *N* = 6 per group. Data are presented as mean ± SEM, one‐way ANOVA, ***p < 0.001.

Because fibrinogen induces BMP activation and NSC‐derived exosomal miR‐128‐3p inhibits the effect of fibrinogen in OPCs, we hypothesized that NSC‐derived exosomal miR‐128‐3p inhibits BMP signaling after MCAO. To further determine the molecular mechanism underlying the therapeutic effect of NSC‐derived exosomal miR‐128‐3p, exosome samples were injected into the striatum of the infarcted hemisphere (Figure 6A). Fibrinogen depletion increased miR‐128‐3p expression (Figure 6B), indicating that fibrinogen targeted miR‐128‐3p. Next, we assessed the expression of ACVR1, which was confirmed by our sequencing analysis as a fibrinogen‐induced target gene of miR‐128‐3p. Pharmacologic depletion of fibrinogen reduced ACVR1 expression, while treatment with NSC‐derived exosomal miR‐128‐3p significantly decreased ACVR1 expression (Figure 6C–E). We also determined Lef1expression, which is known to be regulated by ACVR1. Upon NSC‐derived exosomal miR‐128‐3p treatment, MCAO reduced Lef1 expression (Figure 6D–F). In addition, the levels of P‐Smad1/5 and Id2, transcriptional mediators of BMP signaling, were determined. Treatment with NSC‐derived exosomal miR‐128‐3p robustly reduced P‐Smad1/5 (Figure 6D–G) and Id2 (Figure 6H, I) expression, suggesting that NSC‐derived exosomal miR‐128‐3p suppressed BMP signaling, similar to the pharmacologic reagent ancrod, after MCAO.

### 3.7 NSC‐derived exosomal miR‐128‐3p improves neurological recovery

**FIGURE 7:**
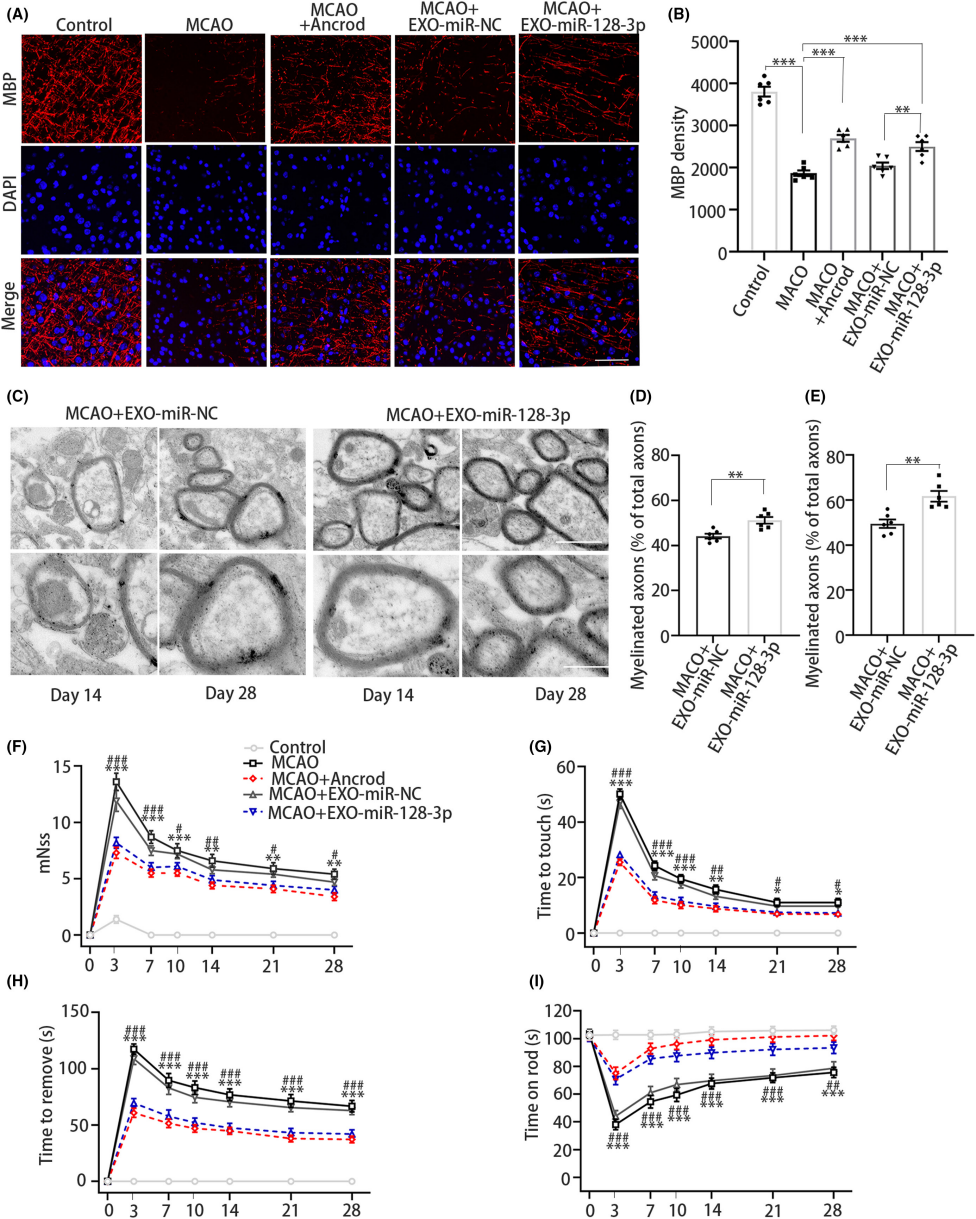
NSC‐derived exosomal miR‐128‐3p improves remyelination and neurological recovery after MCAO. (A) MBP (red) immunostaining in mice. Scale bar, 50 μm. (B) Quantification of MBP immunoreactivity. (C) Transmission electron microscope analysis was performed. Scale bar = 1 μm (top), 0.5 μm (bottom). (D and E) Quantification of the myelinated axons (% of total axons) at 14 and 28 d. *N* = 6 per group. Data are presented as mean ± SEM, one‐way ANOVA or unpaired Student's *t*‐test, **p < 0.01, ***p < 0.001. (F) mNSS. (G) Time to touch of adhesive removal test. (H) Time to remove of adhesive removal test. (I) Rotarod test. *N* = 6 per group. Data are presented as mean ± SEM, Mann–Whitney *U*‐test or unpaired Student's *t*‐test, *p < 0.05, **p < 0.01, ***p < 0.001 vs. control group, ^#^ *p* < 0.05, ^##^ *p* < 0.01, ^###^ *p* < 0.001 vs. MCAO group.

To further elucidate the role of NSC‐derived exosomal miR‐128‐3p in ischemic disease progression, we investigated remyelination, the infarct volume, and functional recovery. NSC‐derived exosomal miR‐128‐3p treatment robustly increased the MBP expression at 7 days (Figure 7A, B) and 28 days (Figure S4A, B), suggesting that exosomal miR‐128‐3p treatment promoted OPC differentiation into MBP^+^ OLs. To determine whether exosomal miR‐128‐3p improves remyelination, we analyzed the myelin structure in the peri‐infarct areas. The number of remyelinated axons was increased in MCAO treated with NSC‐derived exosomal miR‐128‐3p (Figure 7C–E), showing that miR‐128‐3p affected short‐ and long‐term recovery after ischemia.

Next, we investigated whether treatment with NSC‐derived exosomal miR‐128‐3p affects histological outcomes. The infarct volume was significantly reduced in MCAO treated with exosomal miR‐128‐3p (Figure S4C–F), suggesting that brain infarction was alleviated in the early and late stages. There was no significant difference in body weight between NSC‐derived exosomal miR‐128‐3p treated and untreated MCAO groups (Figure S4G). To evaluate neurological function, mNSS assessment, the adhesive removal test, and the rotarod test were performed. Animals in the exosomal miR‐128‐3p treatment group had a lower mNSS from day 3 to 28, indicating that miR‐128‐3p transfection through NSC‐derived exosomes improved neurological recovery (Figure 7F). To exclude mice with no‐qualified sensorimotor function, the animals were evaluated before the adhesive removal test and rotarod test. Exosomal miR‐128‐3p treatment resulted in a significant reduction in the latency to touch and latency to remove the adhesive from day 3 to 28 (Figure 7G, H). In contrast, miR‐128‐3p treatment resulted in a significant increase in the latency to fall off the rod (Figure 7I). Overall, these results suggest that miR‐128‐3p transfection through NSC‐derived exosomes improves neurological recovery, consistent with the effect of the pharmacologic reagent ancrod, after MCAO.

## 4 DISCUSSION

Fibrinogen‐mediated activation of BMP signaling and subsequent inhibition of OPC differentiation and myelin regeneration during the central nervous system pathology have been observed in demyelinating diseases.^6^ Given the relationship between BMP signaling activation and miRNA biogenesis,^35^, ^36^ determining whether miRNAs affect OPC differentiation via BMP signaling is critical. In this study, we verified that fibrinogen was deposited after MCAO and inhibited OPC differentiation into OLs by activating BMP signaling. We also found that miR‐128‐3p targets BMP signaling, isolated exosomes from NSCs, and transferred exosomal miR‐128‐3p to fibrinogen‐treated OPCs. Our results first demonstrated that NSC‐derived exosomal miR‐128‐3p may disturb BMP signaling and promote fibrinogen‐treated OPC maturation. Specifically, NSC‐derived exosomal miR‐128‐3p might promote myelin regeneration and in turn improve functional recovery after MCAO.

We previously investigated whether elevated fibrinogen levels are associated with dependence in ischemic stroke.^2^ Previous studies have demonstrated that fibrinogen activates BMP signaling and inhibits OPC differentiation into OLs in vitro.^6^ This prompted us to explore the mechanism by which fibrinogen prevents remyelination after ischemia. Considering that fibrinogen levels are elevated in stroke patients,^37^, ^38^ while the blood–brain barrier disruption allows blood fibrinogen to enter the central nervous system, we hypothesized that fibrinogen disposition can inhibit remyelination after ischemia. In this study, we found that fibrinogen disposition changed dynamically with the course of MCAO. In addition, we found that fibrinogen inhibited remyelination in vivo and suppressed OPC differentiation into OLs in vitro.

The ability of fibrinogen to inhibit nerve repair has been uncovered, and the associated molecular mechanisms in neurological diseases have been preliminarily eludcidated.^39^ After the blood–brain barrier disruption, fibrinogen enters the brain parenchyma and contributes to neuronal damage and demyelination.^39^, ^40^ In addition to playing a role in coagulation in the central nervous system, fibrinogen directly interacts with nervous cells or their receptors to regulate downstream signaling and nerve cell functions and thus affects repair processes.^1^, ^41^, ^42^ Furthermore, previous studies have indicated that fibrinogen is present both in the extracelluar space and in the cell body and that severe fibrinogen deposition significantly reduces neuronal density, suggesting that elucidating the putative neurotoxicity of fibrinogen and its potential to cause clinical disability is critical.^43^ In addition, accumulating evidence has demonstrated that fibrinogen is sufficient to induce demyelination and that therapeutic depletion of fibrinogen with ancrod enhances remyelination.^6^, ^21^, ^44^ It has been demonstrated that fibrinogen promotes astrogenesis via activation of BMP signaling after ischemia.^4^ We first observed that fibrinogen inhibited OPC differentiation into OLs through BMP signaling and subsequent remyelination after MCAO. In particular, fibrinogen overexpression inhibited remyelination, while therapeutic depletion of fibrinogen reduced this pathologic effect, suggesting that therapeutic strategies that suppress the damaging effects of fibrinogen without affecting its coagulation function are critical after ischemia.

In the last decade, recent studies highlighted the importance of miRNAs as a novel therapeutic target for ischemic stroke.^19^, ^45^, ^46^ To determine the specific changes in miRNAs expression that mediate the inhibitory effect of BMP signaling, we performed whole‐genome microarray analysis of fibrinogen‐treated OPCs. Gene ontology enrichment analysis revealed that miR‐128‐3p inhibited BMP signaling by targeting ACVR1 in fibrinogen‐treated OPCs and promoted MBP expression by reversing the function of fibrinogen.

Although cell‐based therapy was considered to be a potential strategy for treating stroke, the principal mechanism might depend on stem cell‐secreted paracrine factors.^47^ Exosomes act as critical mediators between different neuronal cells and modulate neuronal function.^16^, ^48^ Emerging evidence demonstrated that exosome‐based therapy could amplify the transfer of biomolecules from stem cells to target tissues and have lower immunogenicity.^15^, ^49^ Therefore, exosome‐based therapy might be superior to cell‐based therapy in terms of efficacy, scalability, and translational potential. Several studies have reported that exosomes derived from MSCs can be used to treat stroke models.^50^, ^51^ Intravenous administration of MSC‐derived exosomes post‐stroke improves functional recovery and enhances neurite remodeling, neurogenesis, and angiogenesis.^51^ Recent studies have indicated that the therapeutic effects of MSC‐derived exosomes are mediated by the delivery of miRNAs to regulate cell function.^16^, ^52^ MSC‐derived exosomes enriched with miRNAs robustly improve neurological function and enhance oligodendrogenesis, neurogenesis, and neurite remodeling/neuronal dendrite plasticity after stroke.^11^, ^53^, ^54^ Subsequently, a further study demonstrated that NSC‐derived extracellular vehicles (EVs) improve behavior and mobility in pigs.^13^ In addition, recent studies have shown that transfected NSCs can home to ischemic regions as efficiently as endogenous NSCs, allowing more rapid and better functional reconstruction.^55^ However, no study has shown whether or how NSC‐derived exosomal miRNAs are involved in regulating OPC differentiation after ischemia. In the present study, we demonstrated that NSCs‐derived exosomal miR‐128‐3p induced the OPC differentiation and played a critical role in the recovery process after MCAO. In addition, we identified the potential mechanism underlying miRNA‐mediated remyelination via regulation of the BMP pathway, which was partially activated by fibrinogen overexpression.

BMP signaling is an important negative regulator of OPC differentiation, inhibits endogenous remyelination, and blocks functional recovery.^56^, ^57^ Inhibition of BMP signaling in OPCs promotes myelin formation, and fibrinogen overexpression inhibits OPC differentiation into OLs.^6^ Consistent with previous studies, this study indicated that therapeutic depletion of fibrinogen with ancrod inhibited BMP signaling and promoted final OL maturation. In addition, exosomal miR‐128‐3p could reverse the effect of fibrinogen by targeting ACVR1. Therefore, it is tempting to speculate that miR‐128‐3p induces OPC differentiation into OLs by suppressing BMP signaling and reversing the effect of fibrinogen.

To determine the function of miR‐128‐3p, we applied NSC‐derived exosomes carrying miR‐128‐3p into MCAO by stereotactic injection. Previous studies have demonstrated that exosome‐mediated delivery of miRNAs via intravenous administration protects against ischemic injury and enhances neurogenesis.^58^ Our study first revealed that NSC‐derived exosomal miR‐128‐3p inhibited BMP signaling and promoted remyelination after ischemia. Furthermore, stereotactic injection of NSC‐derived exosomal miR‐128‐3p, which ensures that the intended dose is delivered to the region near the ischemic site and avoids the issue of delivery of a negligible dose to the brain, as achieved by intravenous administration,^59^, ^60^ may reverse the effect of fibrinogen deposition.

There were several limitations in this study. Firstly, neurological recovery after stroke involves nerve regeneration and vascular regeneration, in which OPCs as well as other brain cells participate. Secondly, the biodistribution of EVs in vivo is highly dependent on the cellular origin of the EVs.^61^ Furthermore, previous studies demonstrated that MSC EV treatments trended toward decreasing stroke lesion volume, whereas NSC EVs significantly decreased lesion size and improved neurological function.^12^ However, we did not compare the regenerative property of NSC‐derived exosomal miR‐128‐3p versus MSC‐derived exosomal miR‐128‐3p in this present study. Thirdly, fibrinogen deposition is not the only factor affecting remyelination in the central nervous system. Therefore, OPC differentiation may not be the only promising target for improving neurological function. Further studies analyzing the exosomal miRNAs that regulate other cells during the progression of ischemia, such as endothelial cells and astrocytes, are needed.

## 5 CONCLUSIONS

In summary, fibrinogen deposition plays a critical role in the inhibition of OPC differentiation and remyelination during the progression of ischemia by targeting ACVR1. Specifically, NSC‐derived exosomal miR‐128‐3p inhibits BMP signaling and promotes fibrinogen‐treated OPC differentiation. In addition, demyelination induced by fibrinogen deposition can be antagonized by transfer of miR‐128‐3p by stereotactic injection, which might represent a potential therapeutic target for neural recovery after stroke.

## AUTHOR CONTRIBUTIONS

Yongjun Wang analyzed and interpreted the data in this study. Huiqing Hou performed the majority of the experimental work and was a major contributor in writing the manuscript. Yafei Wang and Lan Yang performed experiments and analyzed data. All authors read and approved the final manuscript.

## CONFLICT OF INTEREST STATEMENT

The authors declare no competing interests.

## Supporting information


Appendix S1

## Data Availability

The microarray data from this publication have been submitted to the GEO database with an accession number GEO: GSE206826 and GSE206950. All data are available from the corresponding author on reasonable request.
