# Supplementary material for: Exosomal miR‐128‐3p reversed fibrinogen‐mediated inhibition of oligodendrocyte progenitor cell differentiation and remyelination after cerebral ischemia
Source: CNS Neurosci Ther. 2023 Feb 8;29(5):1405–22. doi: 10.1111/cns.14113 (PMC10068474; doi:10.1111/cns.14113)
Supplement: Supplementary file 1 — Appendix S1 [file CNS-29-1405-s001.docx]

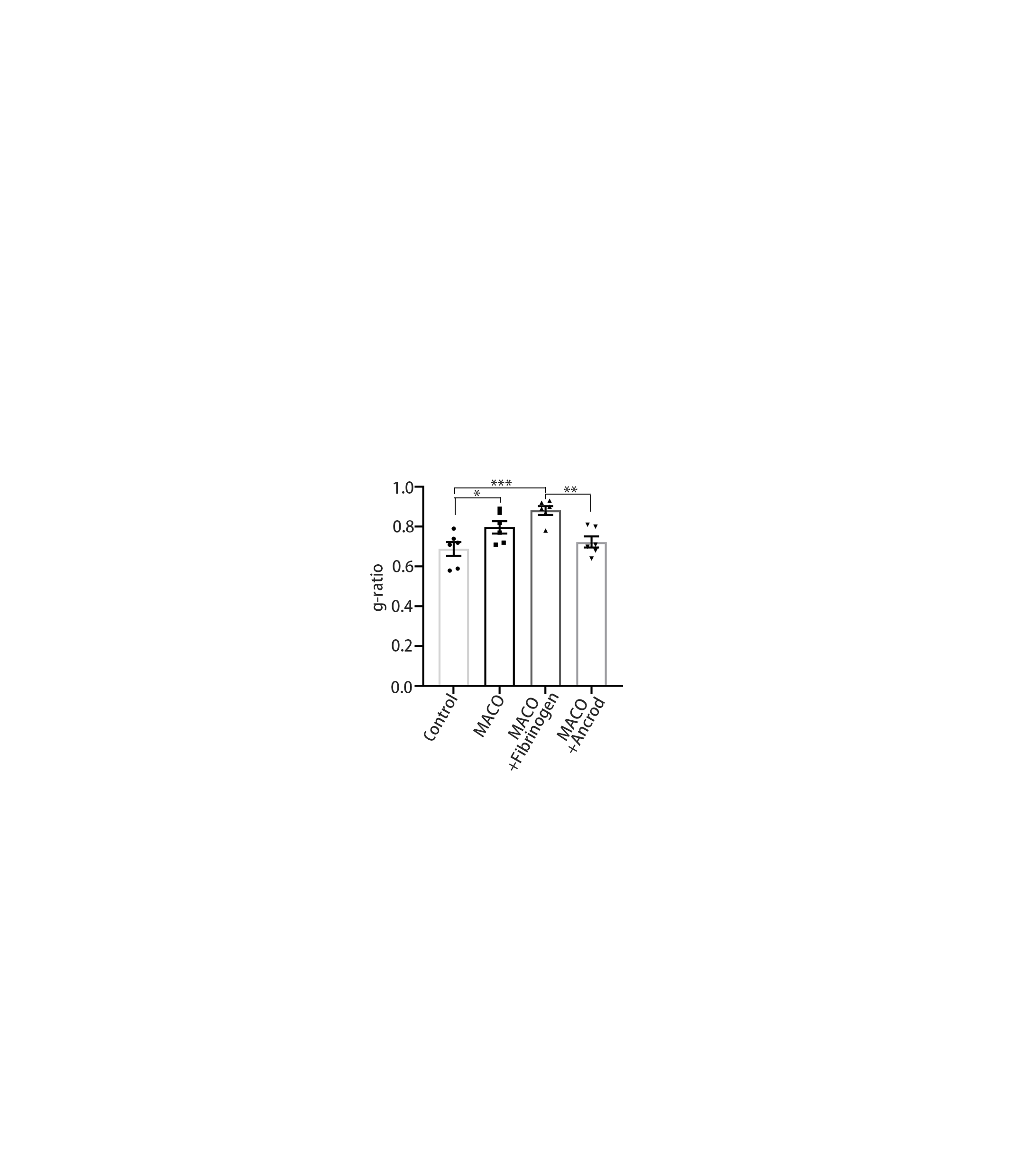


**FIGURE S1** Fibrinogen facilitated the damaging impact on axon. The g-ratio of myelinated axons. N = 6 per group. Data are presented as mean ± SEM, one-way ANOVA, **P*<0.05, ***P*<0.01, ****P*<0.001.


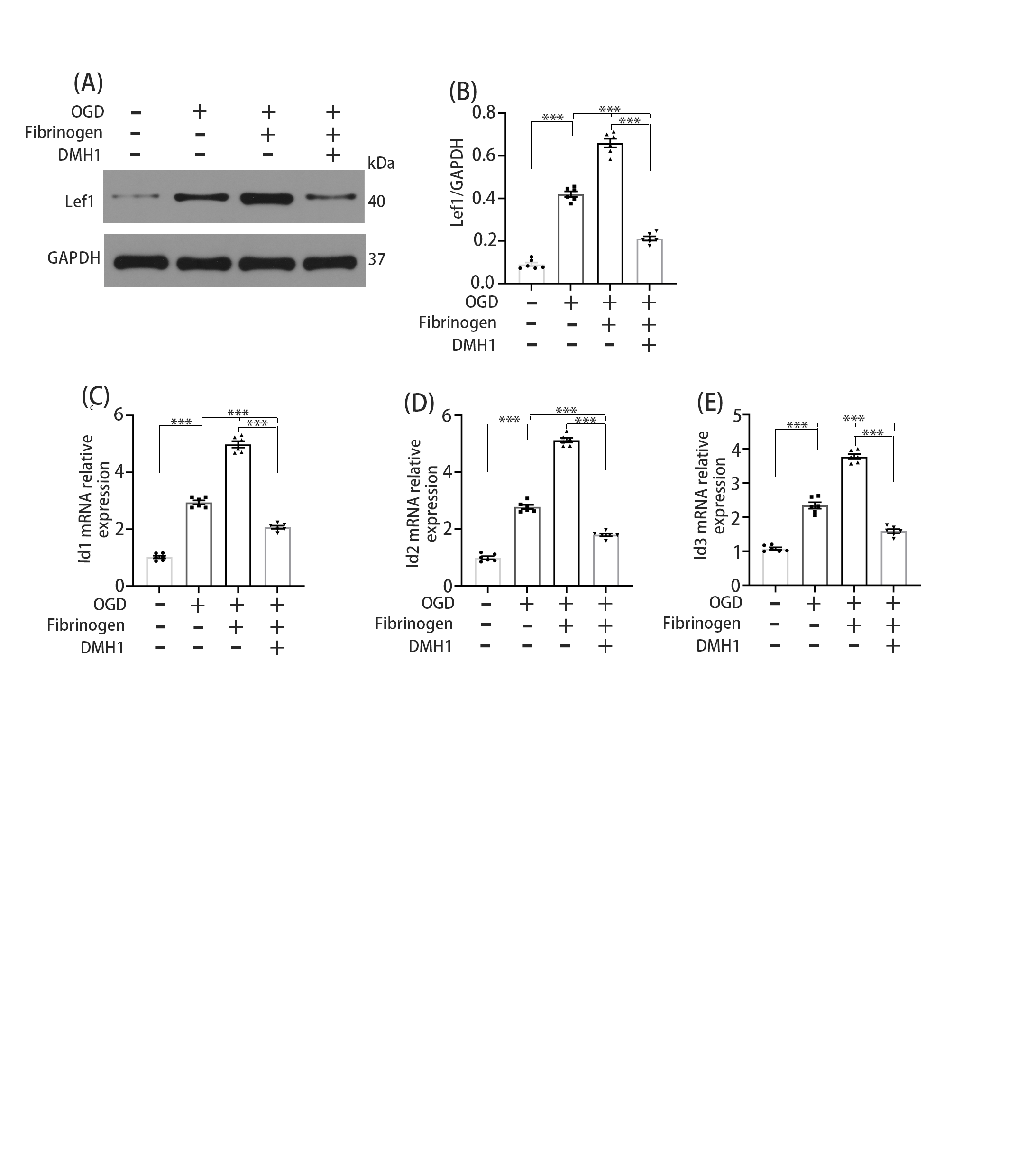


**FIGURE S2** Fibrinogen activates BMP receptor signaling in OGD OPCs. (A) Immunoblot analysis for Lef1 in OGD OPCs. (B) Quantification of Lef1 expression in OGD OPCs. (C-E) Quantitative real-time PCR analysis for Id1, Id2, and Id3 mRNA relative expression in OGD OPCs. N = 6 per group. Data are presented as mean ± SEM, one-way ANOVA, ****P*<0.001.


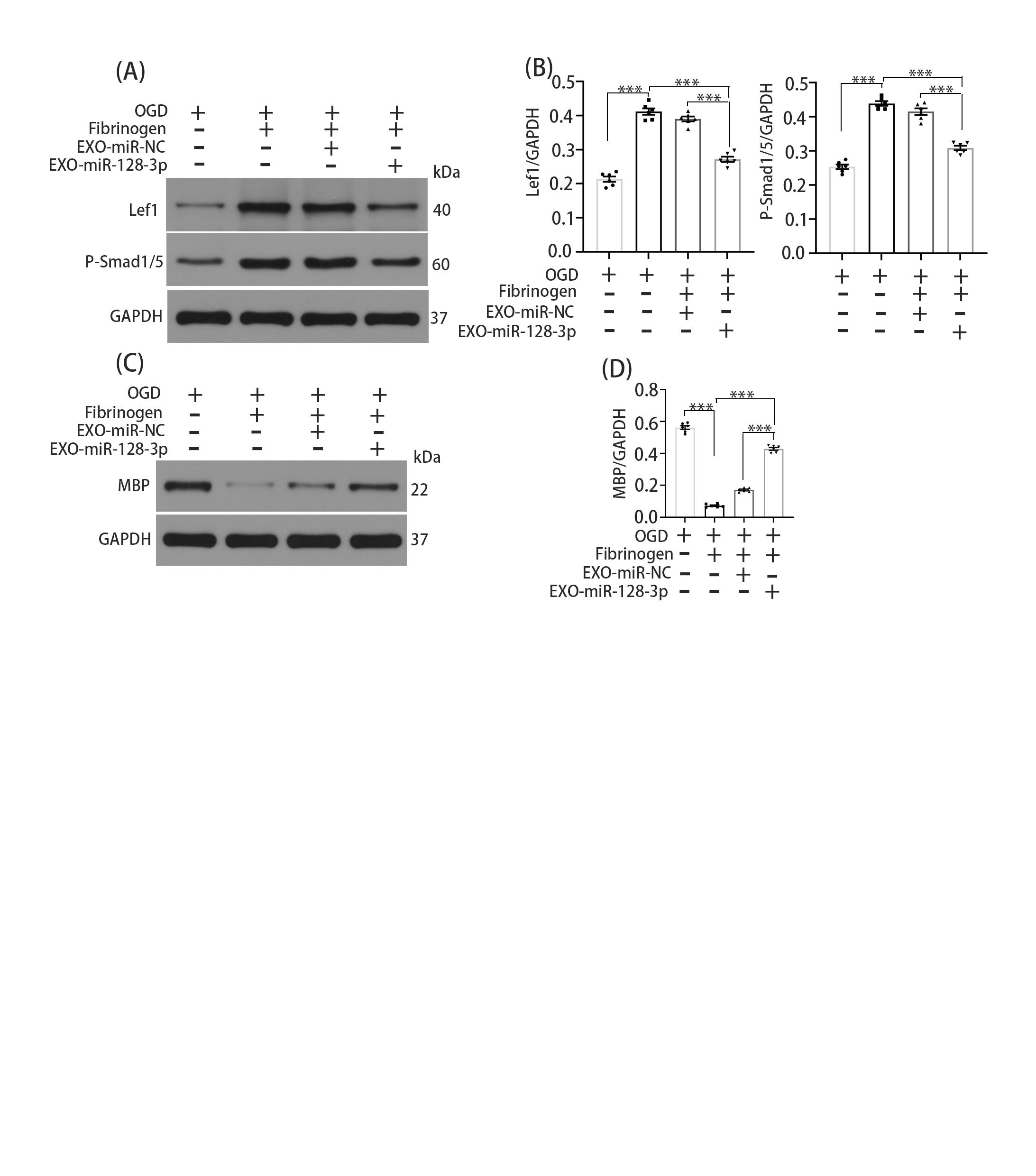


**FIGURE S3** NSC-derived exosomal miR-128-3p promotes the differentiation of OGD OPCs. (A) Immunoblot analysis for Lef1, and P-Smad1/5 in OGD OPCs. (B) Quantification of Lef1, and P-Smad1/5 expression. (C) Immunoblot analysis for MBP in OGD OPCs. (D) Quantification of MBP. N = 6 per group. Data are presented as mean ± SEM, one-way ANOVA, ****P*<0.001.


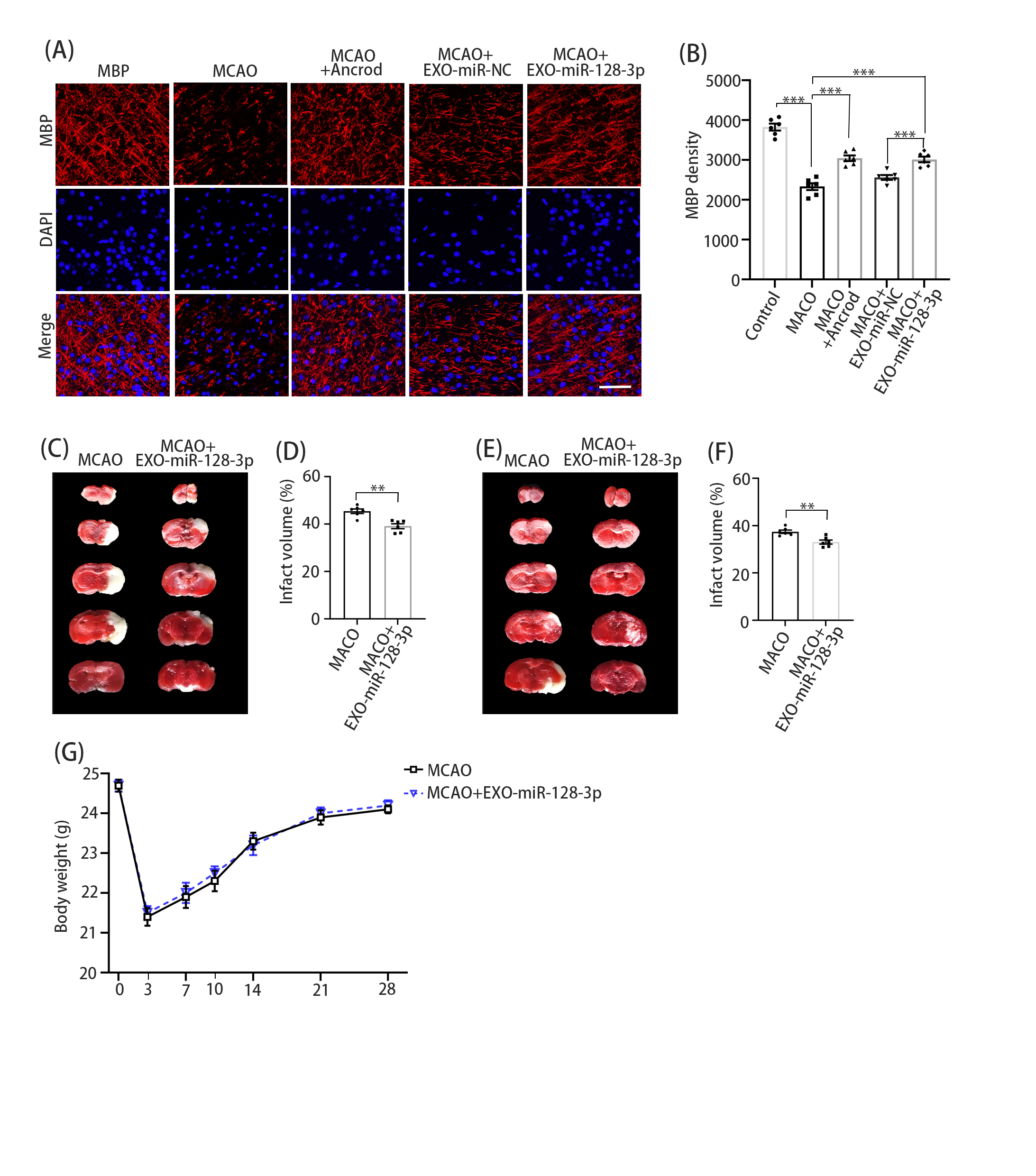


**FIGURE S4** NSC-derived exosomal miR-128-3p modulates OPC differentiation and infarct volume in MCAO. (A) MBP (red) immunostaining at 28 days in mice. Scale bar, 50 µm. (B) Quantification of MBP immunoreactivity. (C) Representative TTC-stained images at day 7 after MCAO. (D) Infarct volume at day 7. (E) Representative TTC-stained images at day 28 after MCAO. (F) Infarct volume at day 28. (G) Body weight. N = 6 mice. Data are presented as mean ± SEM, one-way ANOVA, ***P*<0.01, ****P*<0.001.

**TABLE S1** Primer pairs for quantitative real-time PCR

| Primer | Forward | Reverse |
| --- | --- | --- |
| β-actin | 5’-GAAGAGCTATGAGCTGCCTGAC-3’ | 5’-AGGTCTTTACGGATGTCAACGT-3’ |
| Lef1 | 5’-GCAGCTATCAACCAGATCC-3’ | 5’-GATGTAGGCAGCTGTCATTC-3’ |
| Id1 | 5’-TGGACGAACAGCAGGTGAAC-3’ | 5’-TCTCCACCTTGCTCACTTTGC-3’ |
| Id2 | 5’-CTCCAAGCTCAAGGAACTGG-3’ | 5’-GTGCTGCAGGATTTCCATCT-3’ |
| Id3 | 5’-TGCTACGAGGCGGTGTGCTG-3’ | 5’-AGTGAGCTCAGCTGTCTGGATCGG-3’ |
| MBP | 5’-ATGGCATCACAGAAGAGACCCTCA-3’ | 5’-GATGTAGGCAGCTGTCATTC-3’ |
| U6 | 5’-CTCGCTTCGGCAGCACAT-3’ | 5’-AACGCTTCACGAATTTGCGT-3’ |
| miR-128-3p | 5’-GGTCACAGTGAACCGGTCTCT-3’ | 5’-CTCAACTGGTGTCGTGGAGTC-3’ |
